# Knowledge of teachers concerning food allergies in the primary schools of Edirne city and school facilities for emergent treatment. This study was presented at XVII National Allergy And Clinical Immunology Congress Of Turkey, 3-7 December 2009 Antalya/Turkey

**DOI:** 10.1186/2045-7022-1-S1-O43

**Published:** 2011-08-12

**Authors:** Mehtap Yazýcýoðlu, Pýnar Gökmirza Özdemir, Ülker Öneþ, Hakan Aylanç, Mahir Ceylan, Ufuk Berberoðlu, Mahmut Doðru

**Affiliations:** 1 Trakya University Faculty of Medicine, Pediatric Allergy, Edirne, Turkey; 2 Istanbul University Faculty of Medicine, Pediatric Allergy (Retiree), Istanbul, Turkey; 3 Trakya University Faculty of Medicine, Public Health, Edirne, Turkey

## Objective

Aim of this study is to investigate knowledge of teachers concerning food allergies in the primary schools of Edirne city and school facilities for emergent treatment of severe food reactions.

## Methods

Questionnarrie forms were distributed, Questionnarrie forms were distributed by a pediatrician and a research assistant who tasked in the study project and gathered back after completion of answers in the same day. Before the study, approval of local ethics committee and directorate of national education was taken.

## Results

All of the 237 questionnaire forms that are distributed were gathered back. Ten and half percent of teachers declared that there were students whom have food allergy in their classroom. The most responsible foods in food allergy were reported as egg (19.4%), strawberry (9.4%), chocolate (4.2%) ve tomato (3%). The most commonly observed clinical findings in food allergies were defined as itching (92.4%), rush (76.8%), redness and itching of eyes (65.8), nausea and vomitting (58.2%), abdominal pain (50.6%). It was stated by 41.4% of the teachers that students whom have a life-threatening food reaction are taken to the nearest healthcare unit, 2.5% of the teachers stated that 112 emergency line is called and 0.8% of the teachers stated that parents of the students are notified. The rate of teachers who think that is necessary to give education about food allergy was 95.4%.

## Conclusion

Despite the fact that food allergies may cause life-threatening serious reactions, in our study, the question of existence of individuals to step in to such critical reactions were answered affirmatively in low rate. There is no healthcare stuff in schools to apply immediate intervention in case of food allergies and other life-threatening allergies. We wanted to emphasize that critical reactions due to food allergy may occur during the school time and importance of having knowledge of teachers about this issue.

